# The Effect of Seoritae Extract in Men with Mild to Moderate Lower Urinary Tract Symptoms Suggestive of Benign Prostatic Hyperplasia

**DOI:** 10.1155/2016/1960926

**Published:** 2016-06-13

**Authors:** Woong Jin Bae, Hyo Jung Park, Hye Cheong Koo, Do Ram Kim, U-Syn Ha, Kang Sup Kim, Su Jin Kim, Hyuk Jin Cho, Sung Hoo Hong, Ji Youl Lee, Sung Yeoun Hwang, Sae Woong Kim

**Affiliations:** ^1^Catholic Integrative Medicine Research Institute, College of Medicine, The Catholic University of Korea, Seoul 137-701, Republic of Korea; ^2^Department of Urology, College of Medicine, The Catholic University of Korea, Seoul 137-701, Republic of Korea; ^3^Korea Bio Medical Science Institute, Seoul, Republic of Korea

## Abstract

We evaluated the effects of Seoritae extract (SE) on mild to moderate lower urinary tract symptoms (LUTS) suggestive of benign prostatic hyperplasia (BPH). Seventy-six subjects with mild to moderate LUTS suggestive of BPH were prospectively recruited from the urology outpatient clinic and assigned to either SE (4200 mg or 6 tablets 3 times a day) or matching placebo. The primary outcome variable, the International Prostatic Symptom Score (IPSS), was evaluated at baseline and at 4 and 12 weeks. Postvoid residual volume (PVR), maximum urine flow rate (*Q*_max_), and prostate-specific antigen (PSA) levels were evaluated. IPSSs decreased significantly from baseline to 12 weeks within the SE group. Significant improvements in IPSS voiding scores at 4 and 12 weeks were also observed in the SE group compared to the placebo group. IPSS storage and quality of life scores were also significantly decreased at 12 weeks in the SE group. There was no change in *Q*_max_ or PVR in both groups after 12 weeks. Administration of SE for 12 weeks led to significant improvements in LUTS, and it can be concerned as a reasonable and safe alternative for men with mild to moderate LUTS.

## 1. Introduction

Benign prostatic hyperplasia (BPH), a progressive enlargement of the prostate gland, is an age-related process and highly prevalent among elderly men. The molecular mechanisms associated with the etiology of BPH have not yet been fully elucidated [1]. However, the prevalence of BPH histologically, macroscopically, and clinically increases with age and it is well recognized that testis and aging are risk factors for BPH [2]. Oxidative stress, considered to be a cause of aging, is also thought to play a role in the development of BPH [3, 4]. Hence, it is hypothesized that antioxidant reactions to remove free radicals or inhibit their generation may prevent aging as well as progression of BPH [5].

BPH usually occurs concurrently with lower urinary tract symptoms (LUTS) in aging men and is typically treated with pharmacological therapies, minimally invasive procedures, or surgery. Phytotherapy has recently been used to treat LUTS worldwide because of the many side effects of pharmacological therapies and surgical procedures. Numerous studies have shown that nutritional supplements and herbal medicines can have anti-inflammatory, estrogenic, and antiandrogenic effects owing to decreasing levels of sexual hormones binding globulin; inhibiting growth factor-stimulated proliferation of prostatic cells, lipoxygenase, aromatase, alpha-adrenoceptors, and 5-alpha-reductase; and neutralizing free radicals [6].

Seoritae is a type of black soybean (*Glycine max* (L.) Merr.) grown in Korea. Unlike other black soybeans, the inside of Seoritae has a bluish color. It is a traditional Korean food that also offers health-promoting effects due to its high isoflavone and anthocyanin levels. In our previous study, we proposed that the oxidative stress mechanism is related to BPH progression and that anthocyanin is a potent antioxidant that can decrease prostate volume and prevent BPH progression [7, 8]. We also demonstrated its inhibitory effect on 5-alpha-reductase and showed that the antioxidant properties of isoflavones and anthocyanin in Seoritae extract (SE) could help to prevent BPH occurrence and progression in rats [9].

Thus, we performed a placebo-controlled study to evaluate the clinical effects and safety of SE in human subjects with LUTS suggestive of BPH [10]. We hypothesized that SE would be beneficial in reducing LUTS.

## 2. Materials and Methods

### 2.1. Study Design

This study was conducted at the Catholic University of Korea, Seoul St. Mary's Hospital, in South Korea between December 2013 and August 2014. The study protocol was approved by the institutional review board of the Seoul St. Mary's Hospital of the Catholic University of Korea (KC13HISI0652).

Sample size was estimated from a previous drug study that evaluated International Prostatic Symptom Score (IPSS) differences between two groups as its primary outcome [11]. A mean IPSS difference of 2.9 between the two groups was used to calculate the sample size in this study. A total of 42 subjects in each group were needed for 80% power at a 0.05 significance level, assuming a 15% dropout rate.

Men between 50 and 80 years of age with BPH/LUTS for ≥6 months and IPSS ≥ 8 and ≤19 were recruited if they either had never received treatment (phytotherapeutic agents, alpha-adrenergic blocking agents, or 5-alpha-reductase inhibitors) or had stopped treatment ≥2 weeks prior to enrollment. Exclusion criteria were severe LUTS, defined as IPSS ≥ 20; known hypersensitivity to SE; urinary symptoms due to known causes other than BPH, including urinary tract stones, urethral strictures, urinary tract infections, primary kidney disease, neurogenic bladder, and prostatitis; diagnosed prostate or bladder cancer; previous transurethral prostatectomy; severe cardiovascular disorder; or hepatic disorder. Subjects with uncontrolled psychiatric disorders or senile dementia were also excluded. Subjects with PSA ≥ 4.0 ng/mL were excluded, but those with PSA 4.0–10.0 ng/mL without malignant tumors proven by prostate biopsy were eligible for enrollment.

### 2.2. Study Supplement

The SE used in our study was produced using the following method: Seoritae (200 kg) samples were extracted with 1,600 L of 30% ethanol for 3 hours at 90–100°C. The solution was then filtered twice through a 50 *μ*m and 1 *μ*m filter and concentrated in a vacuum evaporator (60°C) to 70 brix. The residual solvent was removed from the SE with a drying machine for 18 hours at 60°C. The resulting powder was then stored in a plastic bag until use.

Few guidelines are available on the appropriate dose and duration of SE administration for subjects with LUTS [12]. Jang et al. [9] administered SE (SE 1 dose 228 mg/kg and SE 2 dose 457 mg/kg) to rats, and both SE groups had significantly reduced prostate weight and oxidative stress as well as increased apoptosis compared to the BPH-induced control group. Based on previous studies, 2400 mg of SE was administered daily in the current study. The content of isoflavones and anthocyanin in SE was analyzed by high performance liquid chromatography (HPLC) using a Waters 2695 Preparation Module HPLC system with a Waters 996 Photodiode Array Detector at 260 nm or a Waters HPLC system with a 2487 Dual Wavelength Detector set at 520 nm, respectively. The proportions of the isoflavones and anthocyanins in SE are shown in Table 1.

SE tablets (700 mg) were prepared, each containing 400 mg SE. The placebo contained flour with the same dose, shape, and color. Both tablets were produced by HANPOONG Pharm & Foods Co, Ltd., in Jeollabuk-do, South Korea. The release criteria for SE tables are as follows: brown oblong shaped coated tablets with no off-taste and off-odor, the deviation of weight being within 5%, the disintegration time being within 50 minutes, the absence of coliform bacteria and total aflatoxin, the deviation of content of isoflavones being within 20%, the content of lead being less than 3.0 mg/kg, the content of cadmium, total arsenic, and total mercury being less than 1.0 mg/kg, the content of dichloro-diphenyl-trichloroethane and benzene hexachloride being less than 0.1 ppm or 0.2 ppm, respectively, and the content of aldrin, dieldrin, and endrin being less than 0.01 ppm. The tablets were labeled and coded as study supplements. Tablet identity was not revealed to subjects who received the study supplement tablets. The total daily SE dose in this study was 2,400 mg or 6 tablets.

### 2.3. Intervention

Subjects were assigned to receive either placebo or SE (2 tablets 3 times a day) for 12 weeks. All subjects were followed up at 4 and 12 weeks from the start of placebo or SE administration. At the first (screening) visit, a detailed clinical history was taken, including the medical history of present and past diseases and concomitant drug treatments. Prior to receiving the intervention, IPSSs were determined for all subjects. Other objective variables included maximum urine flow rate (*Q*_max_), postvoid residual volume (PVR), digital rectal examination, basic laboratory investigations (hematology, renal function tests, and urine analysis), and serum prostate-specific antigen (PSA) analysis. Compliance and drug-related side effects were assessed at visits 2 and 3 (at 4 and 12 weeks, resp., after starting treatment). Compliance was assessed by counting the number of tablets returned at next study visit.

### 2.4. Main Outcome Measures

The primary outcome measurement is the IPSS questionnaire form which is usually used to assess LUTS at the urology clinic. The IPSS consists of seven questions that assess frequency, nocturia, intermittency, urgency, incomplete emptying, weak stream, and straining with each graded with a score of 0–5. At 12 weeks of treatment, the same efficacy variables were measured for comparison to previous measures. The secondary efficacy outcomes included objective (*Q*_max_, PVR, and PSA) changes from baseline. The safety assessment included subject reports of adverse events and laboratory tests (hematology, clinical biochemistry, and urinalysis). Physical examinations and medical histories were assessed at study entry (baseline), 4 weeks, 12 weeks, and treatment discontinuation.

### 2.5. Statistical Analysis

The data are expressed as the mean (standard error of the mean), with *n* indicating number of experiments. Mean differences between groups were compared by using independent *t*-test and Wilcoxon rank-sum test for normally and nonnormally distributed variables, respectively. *p* values of 0.05 or less were considered statistically significant. All statistical analyses were performed using SAS version 9.3 (SAS Institute, Cary, NC, USA).

## 3. Results

**Figure 1:**
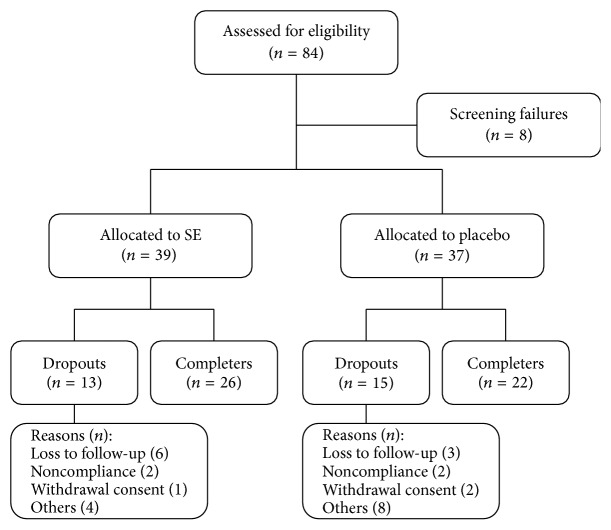
Subject disposition. SE, Seoritae extract.

A total of 84 patients were considered for enrollment. Eight subjects did not meet the inclusion criteria or declined to participate in the study. Seventy-six subjects who met the inclusion and exclusion criteria were assigned to two groups: 39 and 37 subjects in the SE and placebo groups, respectively. After 12 weeks of follow-up, 28 subjects were dropped for noncompliance (*n* = 4) or lost to follow-up (*n* = 9) (Figure 1). Subject demographics and baseline characteristics are shown in Table 2. There were no significant differences between the two groups, except for prostate volume.

### 3.1. Primary Outcome

At the beginning of the trial, IPSSs in the SE and placebo groups were 12.9 ± 3.6 and 13.0 ± 3.7, respectively (*p* = 0.925) (Table 3). IPSSs decreased significantly (*p* < 0.001) from baseline to 12 weeks (10.0 ± 4.4) within the SE group compared to the placebo group (13.0 ± 3.7 at baseline and 14.9 ± 5.7 at 12 weeks) (Table 3). At 12 weeks, the SE group achieved significant (*p* < 0.001) improvements in IPSS voiding (6.1 ± 3.2 versus placebo 8.9 ± 4.1) and storage scores (3.8 ± 2.0 versus placebo 6.0 ± 2.8). IPSS quality of life (QoL) scores decreased significantly from baseline to 12 weeks in the SE group (*p* < 0.001), but not in the placebo group. Differences in IPSS storage scores from baseline to 4 weeks in both groups were not significant. IPSS voiding and storage scores in the placebo group were also decreased from baseline to 12 weeks, but not significantly.

### 3.2. Secondary Outcome

The *Q*_max_ improved slightly in both groups, but the difference between groups was not significant. There was no change in PVR in either group at 12 weeks (Table 4). After 12 weeks, the mean PSA serum levels in the SE group were significantly increased compared with the placebo group. However, the differences of PSA serum levels compared with baseline were 0.2 ± 0.5 ng/mL and −0.1 ± 0.5 ng/mL with the SE group and the placebo group, respectively (*p* = 0.099).

### 3.3. Safety and Tolerability

Among 76 recruited subjects, 5 (6.6%) reported adverse events. There were no severe adverse events in either group during the study period. In the SE group, subjects complained of herpes zoster, constipation, and hematuria (*n* = 1, 2, and 1, resp.), which were tolerable and did not require discontinuation of study supplement tablets. There were no clinically relevant differences in overall AE rates between groups (*p* = 0.359). In addition, there were no significant differences in vital signs and laboratory safety parameters in either group.

## 4. Discussion

The standard treatments for patients with symptomatic BPH include pharmacological therapies (alpha-1-blockers and 5-alpha-reductase inhibitors) and surgery. Although these treatments are most effective for patients with moderate to severe BPH, many patients have complained about their undesired side effects. The most common side effects of alpha-1-blockers include dizziness, postural hypotension, tachycardia, and retrograde ejaculation [13]. The side effects of 5-alpha-reductase inhibitors include erectile dysfunction, loss of libido, and ejaculation disorders [14]. Phytotherapeutic agents have become popular worldwide in patients with mild to moderate symptoms due to the side effects of the standard pharmacological therapies and surgical procedures. The premise of phytotherapy, that plant extracts might be as effective as pharmacologic agents without harmful side effects, makes it attractive to patients who prefer natural remedies [15]. Among phytotherapeutic agents,* Serenoa repens*, also known as saw palmetto, has become one of the 10 top-selling drugs in the United States; many urologists have recommended its use to treat voiding difficulties associated with BPH [16]. A randomized, double-blind, placebo-controlled study showed that administration of saw palmetto led to statistically significant improvements in urinary symptoms in men with LUTS compared with placebo [17]. However, a recent systematic review reported that* Serenoa repens* therapy, even at escalating doses, was not superior to placebo in improvement of symptoms, based on two high-quality clinical trials, one with a follow-up of 6 years [18].

Seoritae is a variety of black soybean with abundant isoflavones and anthocyanin. Anthocyanin is a water-soluble natural pigment that appears as red, purple, and blue in plants and belongs to the flavonoid parent class of molecules. It also acts as a powerful antioxidant with antiangiogenic, anticarcinogenic, and antioxidant effects [19–21]. Jang et al. showed that administration of anthocyanin to rats with induced prostatic hyperplasia resulted in reduced prostate weight and increased apoptosis [7]. Furthermore, anthocyanin significantly decreased apoptotic body count and antioxidant stress in a rat model of varicocele [8]. Isoflavone, a form of phytoestrogen, also possesses biochemical properties that can affect prostate physiology. It is abundant in soy, including primarily genistein and daidzein. Numerous studies have shown that isoflavones affect benign prostatic growth [22, 23] and 5-alpha-reductase activity [24–26]. Most studies have focused on the relationship between isoflavones and the prostate. In our previous study in a rat model of BPH [9], the SE group had significantly decreased prostate weight, oxidative stress, apoptosis, and 5-alpha-reductase. An in vitro study [27] showed that combined soy isoflavones were more efficacious than genistein or daidzein individually in inhibiting prostate epithelial cell growth.

Based on a similar mechanism, we suggested that SE would have an antioxidant effect and increase 5-alpha-reductase-activity due to the synergistic effects of anthocyanin and isoflavones in SE. These results may be the main mechanisms to decrease prostate weight and suppress prostate cell proliferation and these mechanisms may be effective in treating LUTS suggestive of BPH [9]. We found that subjects with BPH in the SE group had improved LUTS based on IPSS. Total IPSS as well as voiding and storage subscores significantly improved from baseline up to 12 weeks in the SE group. After 4 weeks of administration, IPSS was also significantly improved in the SE group compared to the placebo group.

SE did not significantly improve the mean *Q*_max_ or PVR compared with placebo. Our primary goal of treatment with SE is improvement in symptoms, not an increase in *Q*_max_. Therefore, the results of our study suggest that SE may be useful for relief of BPH symptoms although there was no change in *Q*_max_ or PVR after 12 weeks of administration in both groups. Serum PSA levels between the two groups were significantly different, but their change within groups from baseline to 12 weeks was not. A double-blinded, randomized trial with soy isoflavone supplementation conducted by Adams et al. [28] reported increased PSA levels in both groups (soy isoflavone versus no soy isoflavone), but the changes were not statistically significant. This result is also consistent with the previously mentioned study that reported that differences in serum PSA levels in the isoflavones and placebo groups were not significant at the 12th month [29]. There was a high adherence rate (90.7%) with use of the study supplement tablets and no severe adverse events were reported.

This study had several limitations. First, our study was conducted with relatively few samples evaluated over a short period of time. This period was adequate to assess symptom relief but too short to observe any potential reduction of prostate size. For this reason, we did not measure prostate size at 12 weeks. In addition, we identified an unusual discrepancy in subjective outcomes between groups. For example, IPSS voiding and storage scores in the placebo group were also decreased from baseline to 12 weeks, but not significantly. Secondly, there was no restriction on soy food intake, which could be a confounding factor. The possibility for subconscious change in soy intake after entering the study also cannot be ignored. Further clinical studies with larger numbers of subjects and longer study periods are necessary to confirm these findings and determine long-term effects. Restriction or evaluation of dietary soy intake is also necessary in future studies. Thirdly, the baseline imbalance of the prostate volume between the SE and the placebo groups was shown (42.8 ± 13.5 g in SE group and 34.0 ± 13.5 g in placebo group) (Table 2). More enlargement of prostate might be biased toward the null, though there was a weak correlation between IPSS and prostate volume [30–32]. Therefore the difference of IPSS between both groups at 12 weeks might be underestimated. Finally, the SE used in this study was a standardized supplement according to well-established extraction guidelines, but its components could not be completely or chemically defined. Further study to assess its individual efficacy and identify other mechanisms is also necessary. In addition, it should be investigated with large subjects for a longer follow-up.

Administration of SE for 12 weeks led to statistically significant improvements in LUTS compared with administration of a placebo. This study suggests that SE can be concerned as a reasonable and safe alternative for men with mild to moderate LUTS, who choose not to take pharmacological therapies.

## Figures and Tables

**Table 1:** HPLC analysis of isoflavones and anthocyanin content (mg/g) of Seoritae extract. Triplicate samples of 3 lots of the Seoritae extract were analyzed by HPLC.

Content	Seoritae extract		
Daidzin^*∗*^	0.64 ± 0.02	0.64 ± 0.01	0.65 ± 0.02
Genistin^*∗*^	0.68 ± 0.02	0.67 ± 0.01	0.68 ± 0.02
Glycitin^*∗*^	0.10 ± 0.00	0.10 ± 0.00	0.11 ± 0.00
Daidzein	0.02 ± 0.00	0.02 ± 0.00	0.02 ± 0.00
Genistein	0.01 ± 0.00	0.01 ± 0.00	0.01 ± 0.00
Glycitein	0.04 ± 0.00	0.04 ± 0.00	0.04 ± 0.00

Total	1.50 ± 0.04	1.49 ± 0.02	1.51 ± 0.05

Anthocyanin (cyanidin-3-*O-*glucoside)	0.15 ± 0.00	0.15 ± 0.00	0.15 ± 0.00

^*∗*^The content of daidzin, genistin, and glycitin was changed into that of aglycone types of isoflavones using conversion factor of 1/1.6.

Mean ± SD.

**Table 2:** Demographic characteristics and outcome measures at baseline.

	SE group (*n* = 39)	Placebo group (*n* = 37)	Between groups *p* value
Age	64.1 ± 6.3	65.1 ± 7.9	0.647
Weight (kg)	69.1 ± 7.7	71.9 ± 9.6	0.158
Height (cm)	168.7 ± 5.8	169.0 ± 5.6	0.848
Comorbidity	21 (53.8)	27 (72.9)	0.084
Prostate volume (cc)	42.7 ± 13.5	34.0 ± 14.2	0.003
IPSS	12.9 ± 3.6	13.0 ± 3.7	0.925
Voiding score	8.1 ± 3.0	7.9 ± 2.9	0.875
Storage score	4.9 ± 1.9	5.1 ± 2.1	0.953
IPSS QoL	3.3 ± 0.8	3.4 ± 0.8	0.309
*Q*_max_	13.3 ± 5.9	14.5 ± 4.8	0.410
PVR	32.2 ± 29.3	45.7 ± 49.7	0.484
PSA	1.7 ± 1.4	1.4 ± 1.3	0.065

Mean ± SD or *N* (%).

**Table 3:** Mean changes in IPSS total and IPSS subscores from baseline to 12 weeks.

	SE group	Placebo group	Between groups *p* value
Baseline			
IPSS	12.9 ± 3.6	13.0 ± 3.7	0.925
Voiding score	8.1 ± 3.0	7.9 ± 2.9	0.875
Storage score	4.9 ± 1.9	5.1 ± 2.1	0.953
IPSS QoL	3.3 ± 0.8	3.4 ± 0.8	0.309

4 weeks			
IPSS	11.7 ± 4.7^*∗*^	13.8 ± 5.2	0.080
Voiding score	7.2 ± 3.2^*∗*^	8.3 ± 3.6	0.231
Storage score	4.5 ± 2.3	5.5 ± 2.7	0.117
IPSS QoL	3.1 ± 0.8	3.5 ± 0.8	0.019

12 weeks			
IPSS	10.0 ± 4.4^*∗*^	14.9 ± 5.7	0.002
Voiding score	6.1 ± 3.2^*∗*^	8.9 ± 4.1	0.001
Storage score	3.8 ± 2.0^*∗*^	6.0 ± 2.8	0.001
IPSS QoL	2.5 ± 1.1^*∗*^	3.5 ± 1.0^*∗*^	0.001

Mean ± SD, ^*∗*^ *p* < 0.05 changes from baseline.

**Table 4:** Results of urological examinations and serum PSA levels at baseline and 12 weeks.

	Seoritae extract (mean ± SD)	Placebo (mean ± SD)	Between groups *p* value
*Q*_max_, mL/s			
Baseline	13.3 ± 5.9	14.5 ± 4.8	0.410
12 weeks	14.3 ± 6.2	16.3 ± 7.1	0.232
Difference	1.01 ± 4.7	1.9 ± 6.4	0.693

PVR, mL			
Baseline	32.2 ± 29.3	45.7 ± 49.7	0.484
12 weeks	43.9 ± 57.6	34.2 ± 47.7	0.457
Difference	11.8 ± 52.9	−11.5 ± 48.4	0.142

PSA, ng/mL			
Baseline	1.7 ± 1.4	1.4 ± 1.3	0.065
12 weeks	1.9 ± 1.8	1.3 ± 1.3	0.012
Difference	0.2 ± 0.5	−0.1 ± 0.5	0.186

*Q*_max_ = maximum flow rate; PVR = postvoid residual.
